# Ligand-Modified Boron-Doped Diamond Surface: DFT Insights into the Electronic Properties of Biofunctionalization

**DOI:** 10.3390/ma12182910

**Published:** 2019-09-09

**Authors:** Bartłomiej Dec, Michał Sobaszek, Andrés Jaramillo-Botero, William Andrew Goddard, Robert Bogdanowicz

**Affiliations:** 1 Faculty of Electronics, Telecommunications and Informatics, Gdansk University of Technology, 11/12 G. Narutowicza St., 80-233 Gdansk, Poland (M.S.) (R.B.); 2 Materials and Process Simulation Center, California Institute of Technology, 1200 East California Blvd., Pasadena, CA 91125, USA (A.J.-B.) (W.A.G.III)

**Keywords:** DFT, boron-doped diamond, electronic structure and density, GGA, PBE, ab-initio, electronic density of states

## Abstract

With the increasing power of computation systems, theoretical calculations provide a means for quick determination of material properties, laying out a research plan, and lowering material development costs. One of the most common is Density Functional Theory (DFT), which allows us to simulate the structure of chemical molecules or crystals and their interaction. In developing a new generation of biosensors, understanding the nature of functional linkers, antibodies, and ligands become essential. In this study, we used DFT to model a bulk boron-doped diamond slab, modified by a functional linker and a surrogate proteins ligand. DTF calculations enable the prediction of electronic transport properties in an electrochemical sensor setup, composed of a boron-doped diamond electrode functionalized by 4-amino benzoic acids and a target surrogated protein-ligand for influenza. Electron conduction pathways and other signatures associated with the detection and measurement of the target analyte are revealed.

## 1. Introduction

Computational power continues to increase significantly year to year, and by today’s standards, it has enabled rapid simulations, before physical experiments. This helps drive the development of high-sensitivity, high-selectivity biosensing electrochemical surfaces [1]. This involves addressing many electronic structure challenges, including surface activation for improved sensing selectivity, determination of the electrochemical windows, optimisation of surface and linker conductivities, and characterisation of analyte conductivities, among others.

Density Functional Theory (DFT) is an approximation to Schrodinger’s electronic wave equation in quantum mechanics that is used to study multi-body electronic-nuclear systems at their ground state, using functionals of the electron density. DFT is considered relatively cheap in comparison to traditional methods such as Hartree-Fock theory [2], because it directly associates a system’s ground state energy with the electron density, thereby significantly reducing the number of degrees of freedom. DFT is a powerful tool for studying nuclear fuel materials [3], electronic structure and optical properties [4], doping [5] and surface functionalization [6,7,8], among many other materials’ properties and phenomena. 

Thin diamond layers are one of the most promising material for developing new electrochemical sensors [9,10,11]. These layers have high chemical resistance, biological compatibility [12] and a wide electrochemical window. These properties have enabled their broader use in electronics, after appropriate doping [13,14,15]. The most popular and well-known dopant for a diamond is boron, which leads to a p-type semiconductor or even semi-metallic behaviour [16,17]. Furthermore, the diamond surface can be easily functionalized via organic compounds to improve their selectivity and sensitivity in biosensors [18,19].

Many authors have used DFT to study surface functionalization, including Maron and Eisenstein [6,7], who investigated ligands in lanthanide chemistry and tried to answer the question about the role of f-electrons in lanthanide-ligand bonds. Boukhvalow [20] have simulated the covalent functionalization of graphene, concluding that the formation of uniform one- or two-sided functionalized graphene is an exception to the typical scenarios of graphene chemistry. Lastly, Jaramillo-Botero et al. [21] characterised non-covalent ligands on graphene nanoribbons for UDP-glucose sensors and showed the ligands do not affect the material’s transmission spectrum. Recently, Tian and Larrson reported on the adhesion energies of important biomolecules at the variously terminated (111) diamond surfaces [22].

In our recent work [23], we show utilisation of DFT to study of the refractive index of BDD resulting with good agreement with experiments carried out by spectroscopic ellipsometry [24]. The measured values were 2.55–3.37 for a wavelength in the range from 200 nm up to 1600 nm, an error less than 2.5% when compared to bulk diamond. Furthermore, we used a DFT calculation to show electrical and optical properties of allylamine [25]. Achieved results show that with proper functionals and pseudopotentials lead to accurate characterisation of bulk materials. 

In this paper, we investigate the energetic stability and electronic properties of heavily boron-doped diamond (BDD) surfaces, modified by biofunctional ligands that attach to the diamond surface through aminobenzoic linkers. The former, considered here as a surrogate for a broad group of target proteins. DFT [26] with a localised basis set approach [27] was used to determine the electronic and transport properties of the BDD bio-surface, along with a generalised gradient approximation (GGA) exchange-correlation energy correction.

## 2. Materials and Methods 

**Figure 1:**
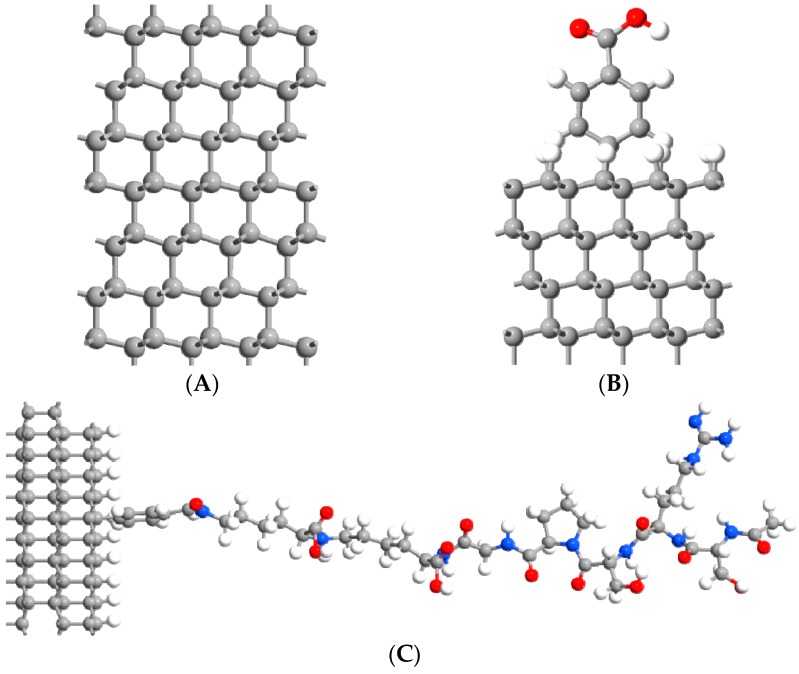
Optimized (4 × 4) model structures: (**A**)—(BDDE) relaxed boron-doped diamond (111); (**B**)—(BDDE-H-ABA)-H-terminated reconstructed boron-doped diamond (111) with adlayer of 4-amino-benzoic acid; (**C**) (BDDE-H-ABA-C3M)-H-terminated reconstructed boron-doped diamond (111) with 4-amino benzoic acid and ligand ending.

We used the QuantumATK software (version 2019.03) [28,29] to calculate the electronic and transport properties of heavily boron-doped diamond electrodes (BDDE) modified by binding of different surface ligands. We assume surface modifications on a pure (111) BDD surface, considering CVD fabricated BDDs are dominated by this crystallographic phase [30] (see Figure 1A–C). The bulk diamond and hydrogen-terminated diamond (111) surfaces were studied for reference and comparison. The diamond was doped with two boron atoms, placed within the third carbon layer from the top surface to provide an acceptor concentration of 3 × 10^21^ cm^−3^ (~2%), as described in our earlier studies [31]. This doping leads to a semiconductor material, with a bandgap of 4.39 eV.

We study the electron-donating and accepting capabilities of a surrogate protein-ligand that is part of the influenza A virus M1 antibody (BDDE-H-ABA-C3M) attached through 4-aminobenzoic acid (4-ABA) to the BDDE surface, as shown in Figure 1C. To characterise this system, we start from a diamond (111) 4 × 4 super-cell slab (Figure 1A). Then we create a hydrogenated diamond surface and remove one of hydrogens from it. Then we attach the 4-ABA linker (Figure 1B), equivalent to an amine cation radical formation of ABA grafted on the carbon-based electrode, and then we covalently attach the protein surrogate ligand to the other end of the 4-ABA (Figure 1C). 

The model system is constructed as a 2-electrode configuration. The electrode region is a repetition of the left or right end of the slab. In all configurations, the left electrode (bottom) has a length of 10.09 Å. In the BDDE, the right electrode has length 10.09 Å. The right electrode length is 5.92 Å. For calculating transmission properties, we use Landauer-Buttiker Formalism (LBF) on an asymmetric electrode configuration. The proposed approach significantly reduces the total number of atoms in the model system, hence cost of calculations. This enables the simulation of a larger ligand surrogate.

Periodic boundary conditions were used in *a* and *b* directions, and the Dirichlet boundary condition was used in the transport direction of the BDDE, *c*. 

Electronic properties simulations were done using the generalised gradient approximation (GGA) with Optimized Norm-Conserving Vanderbilt (SG15) pseudopotentials [32], a linear combination of atomic orbitals [33], a Perdew-Burke-Ernzerhof (PBE) [34] exchange-correlation correction with an energy cut-off of 185 Hartree. Calculations were carried out with k-point sampling at 3x3x101 points in Monkhorst-Pack grid [35]. For optimal comparison of electrical properties, all the slabs have the same A and B cell size.

For the convergence criteria of electronic-state calculations, the total energy change was set to less than 1.0 × 10^−5^ eV/atom. Calculations were carried out with k-point sampling at 3 × 3 × 101 points in Monkhorst-Pack grid [35]. For optimal comparison of electrical properties, all the slabs have the same A and B cell size. 

Calculations of atomic forces used the LCAO local density approximation (LDA) with Fritz-Haber-Institute (FHI) [36] pseudopotentials and the DoubleZetaPolarized (DPZ) basis set. For optimisation of atom positions, we used the Limited Memory Broyden–Fletcher–Goldfarb–Shanno (LBFGS) optimisation algorithm [37]. The convergence of atomic forces was less than 0.01 eVÅ^−1^ with atomic energy change less than 1.0 × 10^−5^ eV/atom. Additionally, a second run with GGA and DFT-D3 dispersion correction included Van der Walls interactions, which are essential in calculating interactions between such small molecules. 

The GGA-PBE algorithm was chosen to describe the electronic structure of BDD [38,39], while the delta-test described by Lejaeghre et al. [40] was used to select the individual atomic potentials, summarised in Table 1. The delta-test results compare the different implementation of pseudopotentials in Quantum ATK to state of the art all-electron pseudopotentials used in WIEN2K code [41].

The SG15 and the DZP pseudopotentials lead to significant differences in the delta-tests [42]. The SG15 medium basis set (with ×2 times higher computational costs when compared to FHI-DZP), results in improved accuracy of almost a factor of 2 in the delta-tests. Further improvement was possible using the SG15 High basis, nearly five times better delta-test (six times higher computational time cost than FHI-DZP, and significantly higher storage requirements). Consequently, we selected SG15-medium as the best cost-performance option for all the results reported in the following sections.

## 3. Results and Discussion

### 3.1. Electron Density Distribution at the BDDE-Analyte Junction

**Figure 2:**
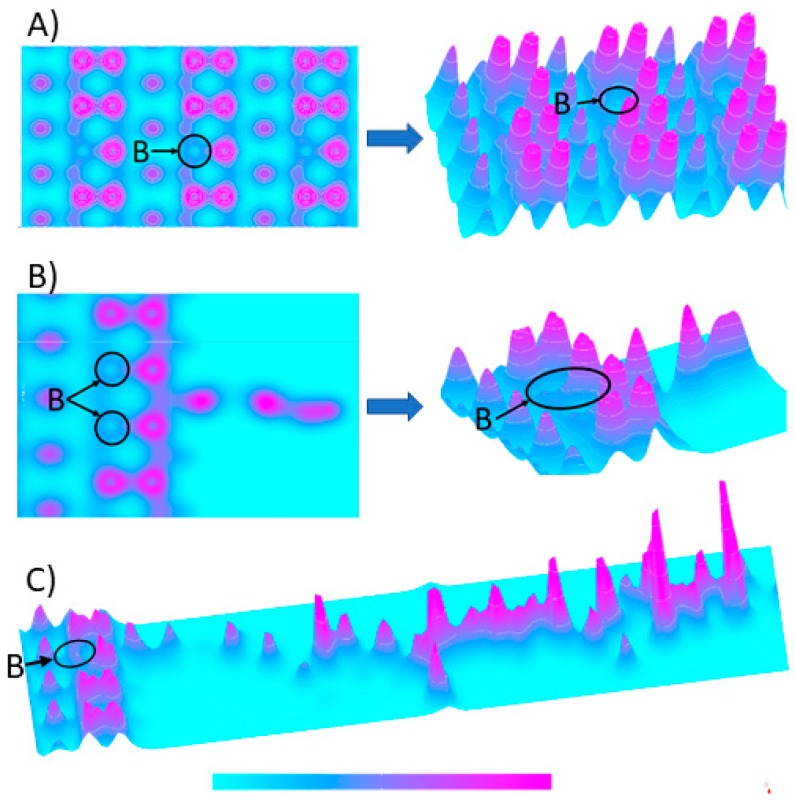
Electron density iso-surfaces: (**A**)—(BDDE) relaxed boron-doped diamond (111) slab; (**B**)—(BDDE-H-ABA)-H-terminated reconstructed boron-doped diamond (111) with adlayer of 4-aminobenzoic linker, (**C**) (BDDE-H-ABA-C3H)—BDDE modified with 4-aminobenzoic linker bonded to C3H ligand of the anti-M1 antibody protein. Azure colour defines electron density close to zero, and the purple describes values up to 1.

The iso-surfaces of the electron density differences obtained from a ground state DFT calculation and a constrained DFT calculation are illustrated in Figure 2. The variation in the electron density in the electrode structures is represented by the violet clouds in 2D and 3D view. The highest probability occurs in an electron close to carbon atoms. The boron doping—acceptor in diamond results in p-type semiconducting [43], which is marked in Figure 2A,B by a blue circular region.

Furthermore, the surface hydrogen termination (dangling-bond) shows a negative electron affinity which will affect band gap [44,45]. The surface functionalization by a 4-aminobenzoic linker (BDDE-H-ABA) does not show significant electron density differences (see Figure 2B) when compared to relaxed BDDE. On the contrary, adding ligand chain effects on electron-densities resulting in higher electron densities at the ligand side. We have observed that BDDE-H-ABA-C3H modification results in the higher electron densities when compared with the pristine BDDE. Thus, the ligand of the anti-M1 antibody act as n-type semiconductor due to functional groups rich in п-bonds [46,47], which make it an electrons source for the BDDE. Once polarised (approx. above 0.5 V), the entire structure works as a P-N junction created by the bio-interface (see Figure 2C) that can be switched and tuned by polarisation [48]. Usually, the electrons within the H-terminated diamond surface are mainly redistributed on the C-H bonds. Nevertheless, electron transfer from the H-terminated diamond surface to the grafted linker and then to ligand is also possible [49].

Takagi et al. [50] showed the mechanism of inducing hole carriers at the H-terminated diamond by physically adsorbed molecules. The electron transfer occurs when the energy level of unoccupied molecular orbitals in an adsorbate molecule is lower than the valence band maximum of H-terminated diamond. The concentration of doped hole carriers depends on the energy level of unoccupied molecular orbital of adsorbate molecules.

DFT computations can give specific information about the electrical properties of junction between the diamond electrode surface and organic compound chosen for its functionalization. This evidence reveals valuable data for designed biosensors based on current-voltage measurements, e.g., cyclic voltammetry in electrochemistry. 

### 3.2. Electron Pathways Study during Device Operation

Electron pathways allow us to gauge the effect of the external potential on the structure’s properties. Electron pathways provide information about what happens during polarisation, e.g. changing character from insulating to semiconducting. To the best of our knowledge this approach is new, and there is lack of scientific literature.

**Figure 3:**
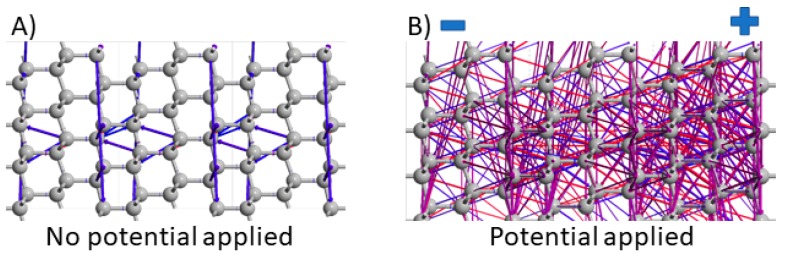
Electron pathways in bulk diamond: (**A**) structure without any external potential across the structure. (**B**) Structure with applied 1V potential across the structure, where on the left side is −0.5V (−), right side 0.5V (+). The 2-terminal device structure was used (attn. electrodes are not shown here for figure readability).

Figure 3 shows a 2-terminal device structure with plotted electron pathways (attn. electrodes are not shown here for figure readability). In the first case (see Figure 3A), the electrons are transferred across bonds pathways where minimal energy is demanded to electron transfer and where are the highest electron densities. When we apply 1V of external potential across the BDDE terminals, the electronic properties shift significantly. Electrons do not travel exclusively along the bonding pathways, but they are also able to hop between neighbouring atoms due to the additional energy supplied. The applied potential spreads across the entire structure, in this case, distributed between −0.5V and +0.5V at the left and right electrodes, respectively. 

**Figure 4:**
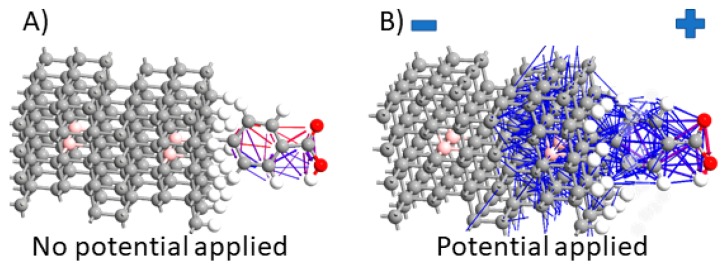
Electron pathways at the diamond surface modified by the attached linker (4-aminobenzoic acid): (**A**) structure without applied potential along C direction (from left to right), (**B**) structure with applied potential −1V, (−0.5V on the left (−), 0.5V on the right side (+)). The 2-terminal device structure was used (attn. electrodes are not shown here for figure readability).

BDDE surface functionalization with the amino-benzoic linker (BDDE-H-ABA) results in two specific electronic conduction pathways (see Figure 4), including one in which electrons travel around the benzene ring via the C-C bonds and another in which the exhibit a hopping-like behaviour directly from the C atoms in the benzene ring to O atoms in the COOH group, as shown in Figure 4A. The polarised structure of BDDE-H-ABA shown in Figure 4B further demonstrates evenly distributed pathways across the 4-ABA and the top BDDE surface layers (attn. electrodes are not shown here for figure readability). The 1 V external potential creates different electron conduction pathways, even from surface (BDDE) H atoms to H atoms located in the benzene ring. This provides evidence on electronic conduction through the 4-ABA linker.

**Figure 5:**
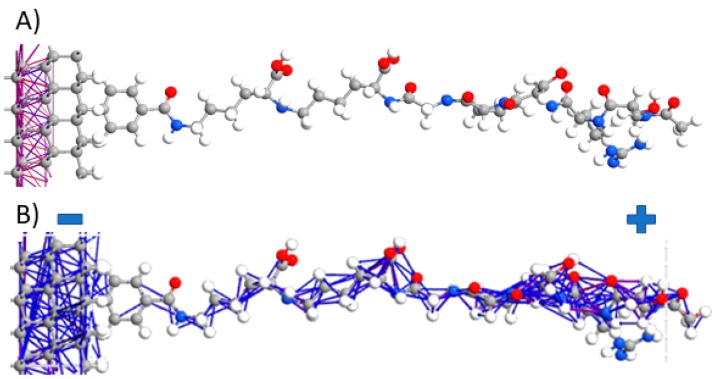
Electron pathways at the diamond surface with attached ligand through linker: (**A**) structure not polarised with potential, (**B**) structure polarised with −1V potential (−0.5V on the left (−) and 0.5V on the right side (+) of the slab). The 2-terminal device structure was used (attn. electrodes are not shown here for figure readability).

Figure 5A of the BDDE-H-ABA-C3H slab shows that electrons prefer the left electrode (−). The right side (+), just minor electron pathways. This fact unambiguously shows that the ligand donates electrons to BDDE suppressing them from the right electrode. The polarised BDDE-H-ABA-C3M structure electron pathways show partial electron tunnelling occurs across the organic molecules and increased conductivity along the BDDE surface. This suggests that the nature of ligand varies thanks to electron tunnelling through structure and reveals a semiconducting behaviour. This effect is illustrated in Figure 5B. Cross-sectional electron tunnelling in Figure 5B exhibits efficient transport along protein bio-interface required for effective biosensing effect [51]. It should be also stated that DFT provides a reliable description of the intramolecular interactions of organic molecules ranging from small to relatively large. Though, calculating the vibrational modes and charge pathways in an organic molecular crystal is a much more complicated because conventional first-principles methods sometimes fail to describe weak intermolecular interactions adequately causing an unphysical expansion of the unit cell during geometry optimisations [48].

### 3.3. Device—Density of States

**Figure 6:**
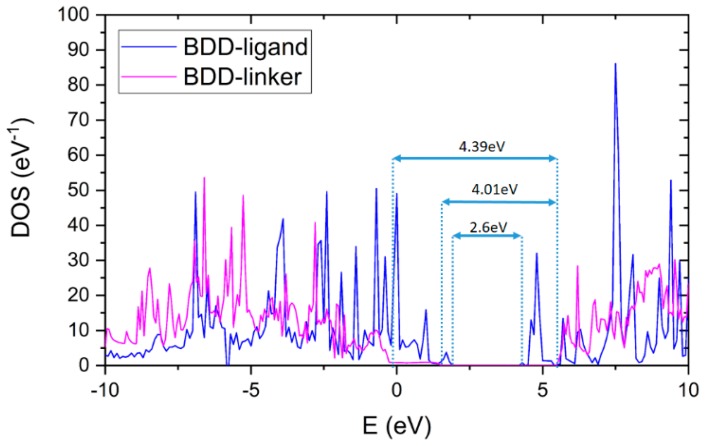
The density of states spectra for BDDE-H-ABA and BDD-H-ABA-C3M with additionally added BDD band gap for change comparison.

The total density of states (DOS) spectra for specific modified diamond structures are shown in Figure 6. It compares the bulk boron-doped diamond (BDD), and the pure diamond electrode surfaces modified by ad-layers BDDE-H-ABA and BDD-H-ABA-C3M. Similar DOS spectra indicating the formation of B-related acceptor impurity band were reported by Ashcheulov et al. [50] by using B dimer supercells configuration or by Wang at el. [52] applying boron substituted doping. Apparently, the Monte Carlo method was applied to estimate band gap of semiconducting boron-doped diamond [53,54]. The band structure and density of states in bulk diamond was simulated by full-band Monte Carlo code for electrons based on the empirical pseudopotential band calculation [54] revealing similar behaviour to DOS presented in Figure 6. Next, Watanabe at al. [53] computed transport properties of hot electrons in bulk diamond utilizing Monte Carlo. They demonstrated band gap energy of 5.47 eV close to experimental values making it competitive for DFT methods but just in case of a bulk diamond, not organic-inorganic interfaces. The deep boron levels could be observed in the DOS of BDDE within the gap, located in the range of 0.0–0.38 eV from the top of the valence band with peak located at 0.1 eV. This is in excellent agreement with other works [55] and the experimental results of 0.365 eV [56].

The BDDE functionalization by ABA and C3M results in the tuning of E_F_ and vacuum level as observed in Figure 6. Accordingly, the band tail states are localised as reported by Dai et al. [45]. In case when there are more electrons at the BDDE than the adsorbate could accept then the electron transfer from BDDE to ABA-C3M occurs, inducing the holes in localized states of the band tail with a valence band bending in the surface layer as revealed by Dai et al. [45]. In such a case, band gap for BDDE-H-ABA and BDDE-H-ABA-C3M was shortened down to 4.01 eV and 2.6 eV respectively. The modification of the diamond surface by ABA and C3M shifts the vacuum level decreasing band gap, contrary to pure hydrogen termination BDDE where surface states are removed from the inside of the band gap [57].

For comparison, the bare BDD results here in the band gap of 4.39 eV. Computed band-gap of BDDE is in good agreement with previous work found in the literature [58,59], where GGA-PBE was applied. Thus, GGA-PBE could be utilized as one of the most cost-effective formalisms used for comparative boron-doped diamond interfaces band gap estimation. Therefore, we have applied here GGA-PBE for cost-effective qualitative trend comparisons, showing a band gap shift during the sequential steps of BDDE surface modification. 

Nevertheless, to achieve better fitting to the experiment, you have to use semi-empirical corrections or hybrid functionals [60,61,62]. Hybrid functionals B3LYP, B3PW91, and HSE06, are computationally expensive and resource-consuming [63], and when it comes to empirical corrections they do not behave the same for each system and must be treated individually, which cannot be applied directly to the examined organic interface which have large vacuum area. Reason for that is that correction parameter is calculated with self-consistency algorithm which calculates integral over the whole unit cell with vacuum included. This vacuum is responsible for causing incorrect or even diverging results [60]. 

Next, Rivero et al. [57] investigated surface properties of hydrogenated diamond in the presence of adsorbates by B3LYP and PBE showing that B3LYP provides much larger band gaps than PBE. O’Donnell et al. [64] also deliberated that the DFT computed band gap of metallic surface is not convincing due to its limitations. They predicted, that bulk band gap of O-terminated diamond was 4.1 eV using GGA, while the value reaches 5.5 eV [65,66]. They proposed to apply scissor correction to GGA eigenvalues (rigid shift of unoccupied states) to achieve an agreement with both experiment and higher levels of theory in the case of diamond [67].

## 4. Conclusions

We show the use of DFT as a powerful tool for *ab-initio* predictions of electrical transport properties in the design of biofunctionalized boron-doped diamond electrodes. The simulation of BDDE and its surface functionalization via 4-amino benzoic acid, and the ligation of a protein surrogate (anti-M1 for influenza) shows that BDD**E**-H-ABA-C3M behaves like P-N junction, the availability of sufficient electron transport pathways and local density of states enable electronic recognition of analytes under externally applied electric fields, a band gap drop from 4.4 eV to 4.01 eV for BDDE to BDDE-ABA, and down to 2.6 eV for BDD**E**-H-ABA-C3M. Furthermore, by monitoring progress in functionalization, either optically or electrically (changing the band gap), we can optimise the conductivity of the sensor and search for optimal treatment. As shown here, DFT can be used to characterise, screen, and optimise the electronic transport properties of electrochemical bio-sensing devices.

## Figures and Tables

**Table 1:** Comparison of delta-test results for pseudopotentials and basis set with chosen atoms that were used in the investigated system.

Atom Type	SG15 (Medium) [meV]	SG15 (High) [meV]	FHI (DPZ) [meV]
Boron	1.65	1.88	3.67
Carbon	0.66	2.80	5.89
Hydrogen	0.96	0.19	0.54
Nitrogen	6.57	1.04	2.45
Oxygen	9.37	1.68	24.28
